# NK cells and CD8+ T cells cooperate to improve therapeutic responses in melanoma treated with interleukin-2 (IL-2) and CTLA-4 blockade

**DOI:** 10.1186/s40425-015-0063-3

**Published:** 2015-05-19

**Authors:** Frederick J Kohlhapp, Joseph R Broucek, Tasha Hughes, Erica J Huelsmann, Jevgenijs Lusciks, Janet P Zayas, Hubert Dolubizno, Vidyaratna A Fleetwood, Alisa Grin, Graham E Hill, Joseph L Poshepny, Arman Nabatiyan, Carl E Ruby, Joshua D Snook, Jai S Rudra, Jason M Schenkel, David Masopust, Andrew Zloza, Howard L Kaufman

**Affiliations:** Rutgers Cancer Institute of New Jersey, 195 Little Albany Street Room 2007, New Brunswick, NJ 08901 USA; Department of Immunology/Microbiology, Rush University Medical Center, Chicago, IL 60612 USA; Department of General Surgery, Rush University Medical Center, Chicago, IL 60612 USA; Department of Internal Medicine, Rush University Medical Center, Chicago, IL 60612 USA; Department of Pharmacology & Toxicology and Sealy Center for Vaccine Development, University of Texas Medical Branch, Galveston, TX 77555 USA; Department of Microbiology and Center for Immunology, University of Minnesota Medical School, Minneapolis, MN 55455 USA; Department of Surgery, Rutgers Robert Wood Johnson Medical School, Rutgers, The State University of New Jersey, New Brunswick, NJ 08903 USA

**Keywords:** Immunotherapy, Interleukin-2, CTLA-4, NK cells, CD8+ T cells

## Abstract

**Background:**

Melanoma is one of the few types of cancer with an increasing annual incidence. While a number of immunotherapies for melanoma have been associated with significant clinical benefit, including high-dose IL-2 and cytotoxic T lymphocyte antigen 4 (CTLA-4) blockade, clinical response to either of these single agents has been limited to 11-20% of treated patients. Therefore, in this study, we sought to test the hypothesis that the combination of IL-2 and CTLA-4 blockade could mediate a more profound therapeutic response.

**Methods:**

Here, B6 mice were challenged with poorly immunogenic B16 melanoma on day 0, and treated with CTLA-4 blocking antibody (100 μg/mouse) on days 3, 6, and 9, and IL-2 (100,000 units) twice daily on days 4–8, or both.

**Results:**

A highly significant synergistic effect that delayed tumor growth and prolonged survival was demonstrated with the combination immunotherapy compared to either monotherapy alone. The therapeutic effect of combination immunotherapy was dependent on both CD8+ T and NK cells and co-depletion of these subsets (but not either one alone) abrogated the therapeutic effect. CTLA-4 blockade increased immune cell infiltration (including CD8+ T cells and NK cells) in the tumor and IL-2 reduced the proportion of highly differentiated/exhausted tumor-infiltrating NK cells.

**Conclusions:**

These results have implications for the design of clinical trials in patients with metastatic melanoma and provide new insights into how the immune system may be mediating anti-tumor activity with combination IL-2 and CTLA-4 blockade in melanoma.

**Electronic supplementary material:**

The online version of this article (doi:10.1186/s40425-015-0063-3) contains supplementary material, which is available to authorized users.

## Background

Melanoma is a tumor of melanocytes and is one of the few types of cancer with an increasing annual incidence [1,2]. Significant advances have been made in treating melanoma using targeted therapy and tumor immunotherapy. Targeted therapy is based on directly inhibiting specific intracellular driver mutations that mediate tumor cell proliferation, and randomized clinical trials have demonstrated improvements in overall survival for patients treated with inhibitors of mutated BRAF and MEK, members of the Ras-Raf-MEK-ERK mitogen-activated protein kinase signaling pathway [3-5]. The benefits in survival are complicated by a nearly universal emergence of drug resistance and tumor recurrence [6]. In contrast to targeted therapy, immunotherapy mediates anti-tumor activity indirectly by activating tumor-specific effector lymphocytes [7,8]. In murine models of tumor immunosurveillance, complete elimination of spontaneously arising tumors is possible but depends on several factors, including interferon-gamma, Fas/FasL interactions, perforin, NKG2D, and an intact lymphocyte compartment [9]. Tumor immunotherapy mediates anti-tumor activity by enhancing lymphocyte responses, notably of cytotoxic CD8+ T cells. Immunotherapy has been associated with significant clinical benefit characterized by durable responses in subsets of patients even in the presence of advanced metastatic disease [10,11]. Several agents, including interferon-alpha, high-dose interleukin-2 (IL-2), and checkpoint inhibitors targeting the cytotoxic T lymphocyte antigen 4 (CTLA-4) and programmed cell death 1 (PD-1) receptors have been approved by the FDA for the treatment of metastatic melanoma [12].

IL-2 is a four-bundle, alpha-helical cytokine released by lymphocytes that binds to the high-affinity trimeric IL-2 receptor expressed on activated lymphocytes and natural killer (NK) cells. IL-2 mediates proliferation and differentiation of T, B, and NK cells and promotes clonal expansion following T cell receptor (TCR) recognition of cognate antigen [13]. IL-2 has been associated with objective response rates of 16-20% in patients with metastatic melanoma and responders have durable tumor control with nearly 90% of complete responders free of disease recurrence up to 15 years following treatment [14]. High-dose IL-2 presumably mediates anti-tumor activity through expansion of the CD8+ T cell and NK cell compartments, but it is also known to expand regulatory CD4+ FoxP3+ T cells (Tregs), which can mediate immune suppression and promote peripheral tolerance [15,16]. In fact, low doses of IL-2 have been used to protect against graft-versus-host disease and may abrogate autoimmunity [17,18]. Melanoma patients treated with high-dose IL-2 have shown an expansion of the Treg compartments; however, those patients with a clinical response exhibit a paradoxical decrease in Tregs following treatment [19]. Although the mechanism of this decrease is not understood, blocking the expansion of Tregs could improve tumor immunotherapy with IL-2 by taking advantage of the expansion of CD8+ effector T and NK cells [20].

CTLA-4 is an immunoglobulin-like family co-receptor that is mobilized to the T cell surface following TCR engagement and co-stimulation [21]. Full activation of T cells requires two signals, the first mediated by the TCR upon recognition of antigen in the form of peptide bound to MHC class I and II molecules for CD8+ and CD4+ T cells, respectively. CD28 is a master co-stimulatory receptor that is expressed near the TCR and can be stimulated by B7.1 (CD80) and B7.2 (CD86), usually expressed by antigen-presenting cells during T cell priming [22]. CD28 signals intracellularly and coordinates cell proliferation, cytokine production, differentiation and blocks lymphocyte apoptosis [23]. Following co-stimulation, CTLA-4 is mobilized to the cell surface where it binds with higher affinity to CD80 and CD86, thereby inhibiting lymphocyte effector functions. CTLA-4 thus acts as a T cell checkpoint inhibitor, blocking uncontrolled effector cell activity and likely functions to prevent autoimmunity. Ipilimumab is an IgG1 monoclonal antibody that blocks the interaction of CTLA-4 with its ligand and promotes the activation of T cells [24]. The FDA approved ipilimumab for the treatment of melanoma in 2011 after a randomized clinical trial showed an improvement in overall survival in patients with metastatic melanoma [25]. The objective response rate with ipilimumab was reported to be 10.9%, although when responses occurred they were often quite durable [25,26].

In general, clinical response to single agent therapy (monotherapy) has been limited to a small group of patients, generally in the range of 11-24% [26]. While these agents have shown significant therapeutic activity against a variety of murine tumors, they have demonstrated only limited therapeutic activity against the poorly immunogenic murine B16 melanoma [27-31]. Thus, the B16 model may represent a relevant system for evaluating new treatment approaches and can be used to identify the cellular and molecular mechanisms that result in more meaningful therapeutic activity. The possibility of combining immunotherapy agents has suggested that additive and even synergistic activity may be observed in both murine tumor models and in early phase clinical trials [31,32,2]. In this report, we sought to test the hypothesis that combination of high-dose IL-2 and CTLA-4 blockade could mediate more profound therapeutic activity using the B16 melanoma tumor model. A highly significant synergistic effect on survival was demonstrated, without added toxicity, and this effect was dependent on both CD8+ T and NK cells. These results have implications for the design of clinical trials in patients with metastatic melanoma and provide new insights into how the immune system may be mediating anti-tumor activity with combination IL-2 and CTLA-4 blockade.

## Results and Discussion

### Combination immunotherapy with IL-2 and CTLA-4 blockade results in significantly delayed tumor growth and prolonged survival

**Figure 1:**
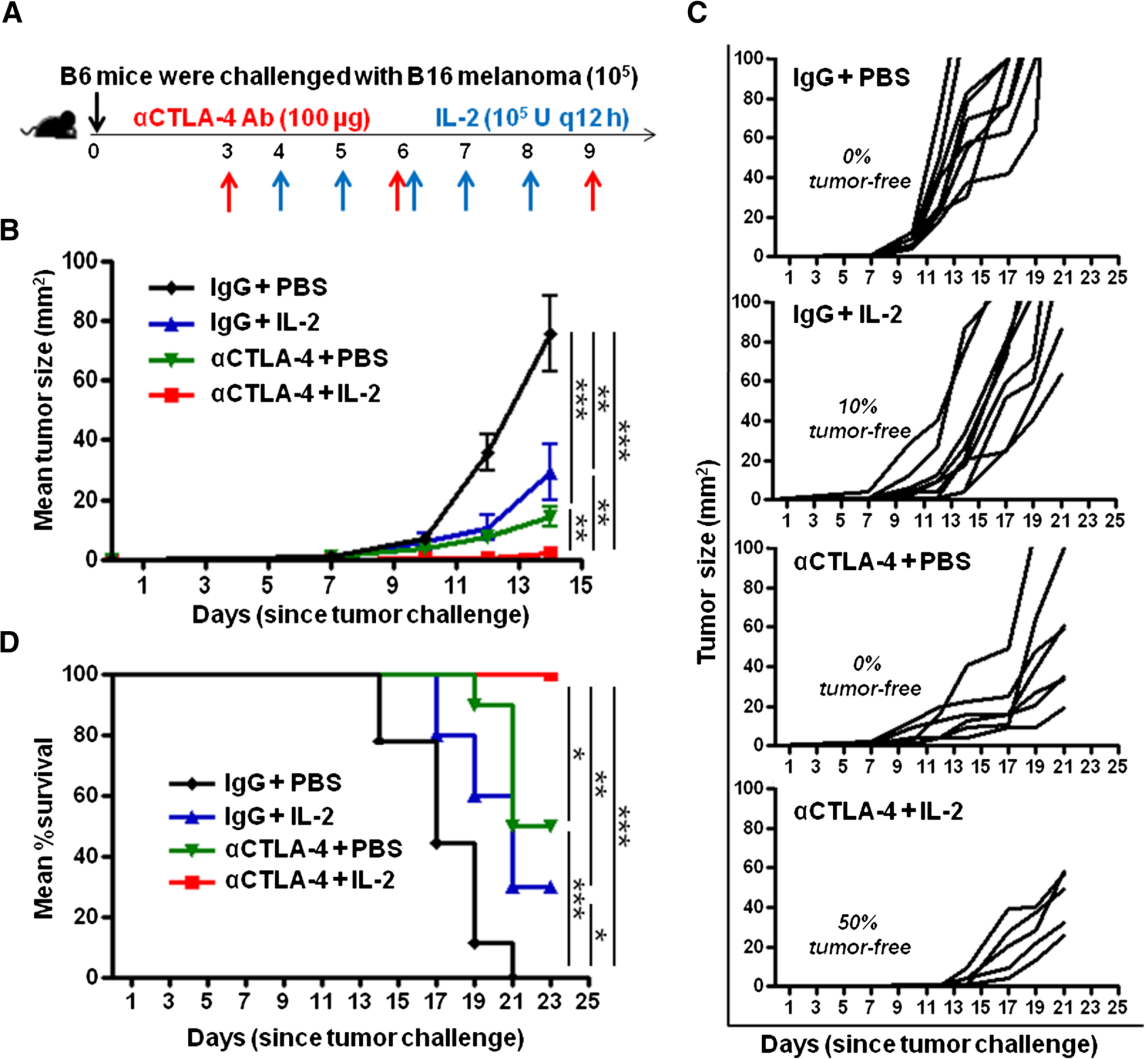
Combination IL-2 and CTLA-4 blockade immunotherapy results in reduced tumor growth and prolonged survival. **(A)** Schematic of the experimental design. **(B)** Cumulative graph of mean tumor size (mm^2^) per group from experiment described in **(A)**. **(C)** Tumor size (mm^2^) of individual mice in each group from experiment described in **(A)**. **(D)** Cumulative graph of mean percent (%) survival per group from experiment described in **(A)**. Seven to ten mice were included in each group. Graphs represent one experiment of six conducted with similar results. *P < 0.05, **P < 0.001, ***P < 0.001, ns = not significant.

IL-2 therapy and CTLA-4 blockade have individually been shown to improve anti-tumor responses and are approved as monotherapies for the treatment of metastatic melanoma [12,14,25]. Since each of these monotherapies may work through a unique mechanism, we explored the effect of combination IL-2 and CTLA-4 blockade immunotherapy using the murine B16-F10 (B16) melanoma model. Specifically, we challenged mice with B16 (1–1.2 x 10^5^ cells by intradermal injection) and treated with CTLA-4 blockade (αCTLA-4; 100 μg via intraperitoneal injection [i.p.] on days 3, 6 and 9 after tumor challenge) only, IL-2 (100,000 units i.p every 12 hours on days 4–8 post tumor challenge) only, or the combination IL-2 and CTLA-4 blockade. We designed the treatment regimen to correspond with the manner in which these monotherapies are utilized in the clinical setting (Figure 1A). We found that both monotherapies, IL-2 and CTLA-4 blockade individually delayed tumor growth compared to the control (IgG + PBS) treatment (29 mm^2^ and 14 mm^2^ versus 76 mm^2^; p < 0.01 and p < 0.001, respectively, at day 14) (Figure 1B, C) and prolonged survival (30% and 50% versus 0%; p < 0.05 and p < 0.001, respectively, at day 23) (Figure 1D). Combination IL-2 and CTLA-4 blockade resulted in significantly delayed tumor growth compared to IL-2 only or CTLA-4 blockade only (2 mm^2^ versus 29 mm^2^ [p < 0.01] and 14 mm^2^ [p < 0.01], respectively, at day 14) and significantly prolonged survival (100% versus 30% [p < 0.01] and 50% [p < 0.05], respectively, at day 23) (Figure 1B-D). No added toxicity was detected with combination IL-2 and CTLA-4 blockade compared to either treatment alone (Table 1). These findings demonstrate an augmented anti-tumor effect with combination IL-2 and CTLA-4 blockade immunotherapy compared to either monotherapy alone against established, poorly immunogenic melanoma.

**Table 1:** **Chemistry screen IL-2 and of CTLA-4 blockade immunotherapy**

**Treatment**	**Naive**	**IL-2**	**CTLA-4 blockade**	**CTLA-4 blockade + IL-2**	**Reference ranges**
**Alkaline Phosphatase (U/L)**	49	12	56	50	34 - 104
**ALT (U/L)**	40	198	26	63	8 - 40
**AST (U/L)**	160	238	134	123	10 - 45
**Total Bilirubin (mg/dL)**	0.35	0.72	0.44	0.30	0.20 - 0.10
**BUN (mg/dL)**	28	34	36	35	6 - 23
**Creatinine (mg/dL)**	0.17	0.05	0.12	0.2	0.50 - 1.30
**Calcium (mg/dL)**	8.13	6.14	7.86	11.58	8.40 - 10.50
**Glucose (mg/dL)**	58	32	18	27	75 - 200
**Albumin (g/dL)**	3.95	3.79	4.13	3.91	3.50 - 5.70
**Total Protein (g/dL)**	7.17	6.62	7.01	6.80	6.00 - 8.40
**Phosphorus (mg/dL)**	38.1	33	34.6	35.5	2.4 - 4.5

ALT, Alanine Transaminase; AST, Aspartate Aminotransferase; BUN, Blood Urea Nitrogen.

### CTLA-4 blockade promotes immune cell infiltration within the tumor

**Figure 2:**
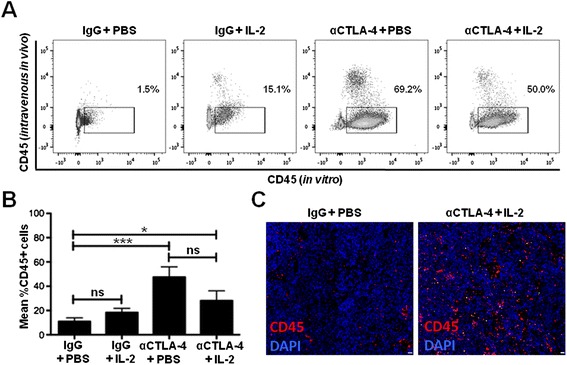
Combination IL-2 and CTLA-4 blockade immunotherapy increases tumor immune infiltration. **(A)** Flow cytometry plots of tumors dissected at day 14 and analyzed by flow cytometry for CD45 expression from the experiment described in Figure 1A. Only tumor-infiltrating lymphocytes were analyzed (as determined by comparison of intravenous CD45 staining compared to *in vitro* CD45 staining). **(B)** Cumulative graph showing mean percent CD45+ T cell infiltration (of live cells) in the tumor from three independent experiments described in **(A)**. **(C)** Representative immunofluorescence microscopy images of CD45 (red) and DAPI (4′,6-diamidino-2-phenylindole; blue) staining of tumors from experiment in **(A)**. Scale bars = 20 microns. Three to five mice were included in each group per experiment. *P < 0.05, ***P < 0.001, ns = not significant.

Immune infiltration within the tumor is a positive prognostic indicator of response to immunotherapy [33,34]. Thus, we assessed immune infiltration of B16 by determining the proportion of CD45+ cells within the tumor following treatment. To distinguish between tumor-resident immune infiltration and immune cells restricted to the vasculature, we intravenously injected anti-CD45 conjugated to a fluorophore three minutes prior to sacrificing the mice. Following tumor dissection and tissue dissociation, we stained cells *in vitro* with anti-CD45 conjugated to a different fluorophore than the one used for intravenous *in vivo* staining. This permitted the identification of tumor-resident leukocytes by flow cytometry (Figure 2A). CTLA-4 blockade alone and in combination with IL-2 promoted CD45+ immune infiltration (among live cells) compared to the control treatment (mean %CD45+ cells in the tumor: 48% and 28% versus 11%, p < 0.001 and p < 0.05, respectively) (Figure 2A,B). No significant difference in immune infiltration was observed between the combination group and the CTLA-4 blockade treatment group (48% versus 28%, respectively, p > 0.05) or between IL-2 alone and the control group (18% versus 11%, respectively, p > 0.05) (Figure 2B). Further, to confirm that the combination immunotherapy enhanced immune infiltration, we used epifluorescence microscopy. This demonstrated that IL-2 and CTLA-4 blockade combination immunotherapy results in augmented CD45+ immune cell infiltration within the tumor (1x10^5^ versus 4x10^4^ CD45+ cells/10^6^ nuclei, respectively, p < 0.05) (Figure 2C). These data demonstrate that CTLA-4 blockade, individually and when combined with IL-2, promotes immune cell infiltration within the tumor microenvironment.

### CTLA-4 blockade results in increased CD8+ T cells in the tumor microenvironment

**Figure 3:**
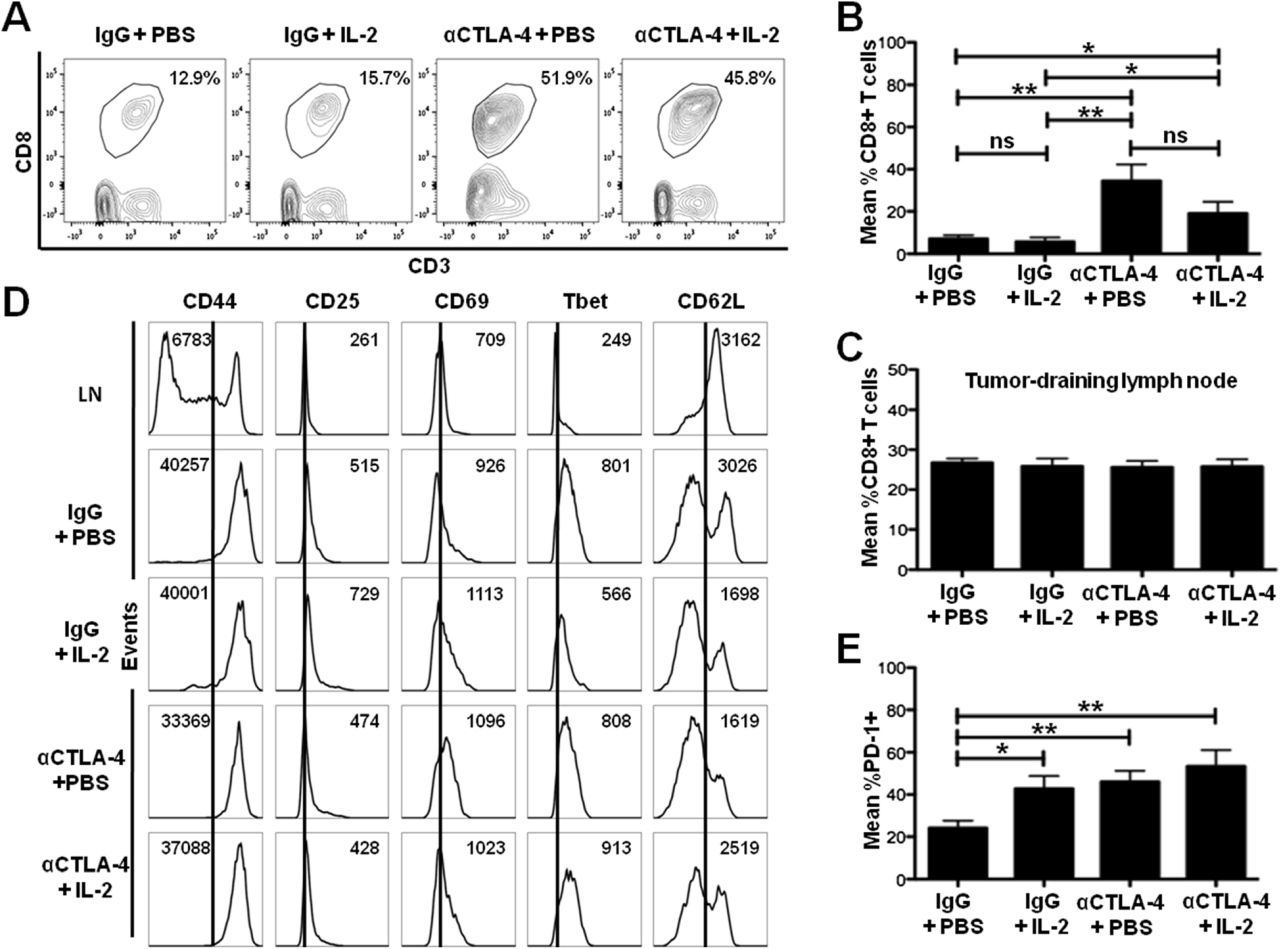
Combination IL-2 and CTLA-4 blockade immunotherapy increases the proportion of tumor-infiltrating CD8+ T cells. **(A)** Flow cytometry plots of tumors dissected at day 14 and analyzed for CD3 + CD8+ T cells (of CD45+ cells within the tumor) by flow cytometry from the experiment described in Figure 1A. **(B)** Cumulative graph showing the mean percent of CD3 + CD8+ T cells from experiment in **(A)**. **(C)** Cumulative graph showing the mean percent of CD3 + CD8+ T cells in the tumor-draining lymph nodes from experiment in **(A)**.** (D)** Representative histograms showing expression of cell markers from CD3 + CD8+ T cells in the tumor and from a representative tumor-draining lymph node (LN). Numbers represent mean fluorescence intensity (MFI). **(E)** Cumulative graph of mean percent of CD3 + CD8+ T cells expressing PD-1 from experiment in **(A)**. Cumulative figures are from at least three independent experiments (with 3–5 mice per group in each experiment). *P < 0.05, ** P < 0.01, ns = not significant.

CD8+ T cells are vital mediators of anti-tumor responses. Therefore, we characterized the tumor immune infiltrate to determine the effect of combination IL-2 and CTLA-4 blockade on CD8+ T cells. CTLA-4 blockade alone and in combination with IL-2 resulted in an increased proportion of CD8+ T cells among the immune cell infiltrate (CD45+ cells) of the tumor compared to the control group (35% and 19% versus 7%, p < 0.01 and p < 0.05, respectively) (Figure 3A,B). No significant difference in immune infiltration was observed between the combination group and the CTLA-4 blockade only treatment group (19% versus 34%, respectively, p > 0.05) or between IL-2 alone and the control group (6% versus 7%, respectively, p > 0.05) (Figure 3A, B). These data suggest that CTLA-4 blockade may be the primary driver of CD8+ T cell tumor infiltration after CTLA-4 blockade with or without IL-2 immunotherapy.

To determine whether the increased proportion of CD8+ T cells in the tumor microenvironment was the result of an overall systemic increase in CD8+ T cells, we analyzed the tumor-draining (inguinal) lymph nodes and spleen for CD8+ T cell numbers. No increase was observed in the proportion of CD8+ T cells within the tumor-draining lymph nodes as a result of IL-2 alone, CTLA-4 blockade alone, or combination IL-2 and CTLA-4 blockade compared to the control treatment (in all groups CD8+ T cells constituted approximately 27% of CD45+ cells, p > 0.05 for all comparisons) (Figure 3C). Similarly, no differences in CD8+ T cells amongst the groups were seen in the spleen (data not shown). This demonstrates that the increased proportion of tumor-infiltrating CD8+ T cells is not the result of a systemic increase in peripheral CD8+ T cells, but rather changes specific to the tumor microenvironment.

To determine whether the activation status of CD8+ T cells correlates with the anti-tumor immune responses we observed (Figure 1B,C), we determined the expression of CD8+ T cell activation markers. Tumor-infiltrating CD8+ T cells showed an activated phenotype (with increased CD44, CD25, CD69, and Tbet expression and decreased CD62L expression) compared to CD8+ T cells in the tumor-draining lymph nodes (Figure 3D). However, we observed no differences in these activation markers when comparing the monotherapies (IL-2 or CTLA-4 blockade) or the combination IL-2 and CTLA-4 blockade immunotherapy to the control group (IgG + PBS) (Figure 3D). Since PD-1 is known to be upregulated during T cell activation, we likewise determined its expression on tumor-infiltrating CD8+ T cells. IL-2 and CTLA-4 blockade monotherapies as well as the combination IL-2 and CTLA-4 blockade immunotherapy resulted in an increased proportion of CD8+ T cells expressing PD-1 (among all CD8+ T cells) within the tumor (46%, 43%, 53% versus 24%, p < 0.01, p < 0.05, and p < 0.01, respectively) (Figure 3E). However, no differences in PD-1 expression between the combination immunotherapy and the CTLA-4 blockade and IL-2 monotherapies (53% versus 46% and 43%, p > 0.05 for both, respectively) were detected. These data demonstrate the activation status of CD8+ T cells does not correlate with the improved anti-tumor immune responses we observed for combination IL-2 and CTLA-4 blockade immunotherapy.

### Combination CTLA-4 blockade and IL-2 immunotherapy increases regulatory T cells within the tumor immune infiltrate

**Figure 4:**
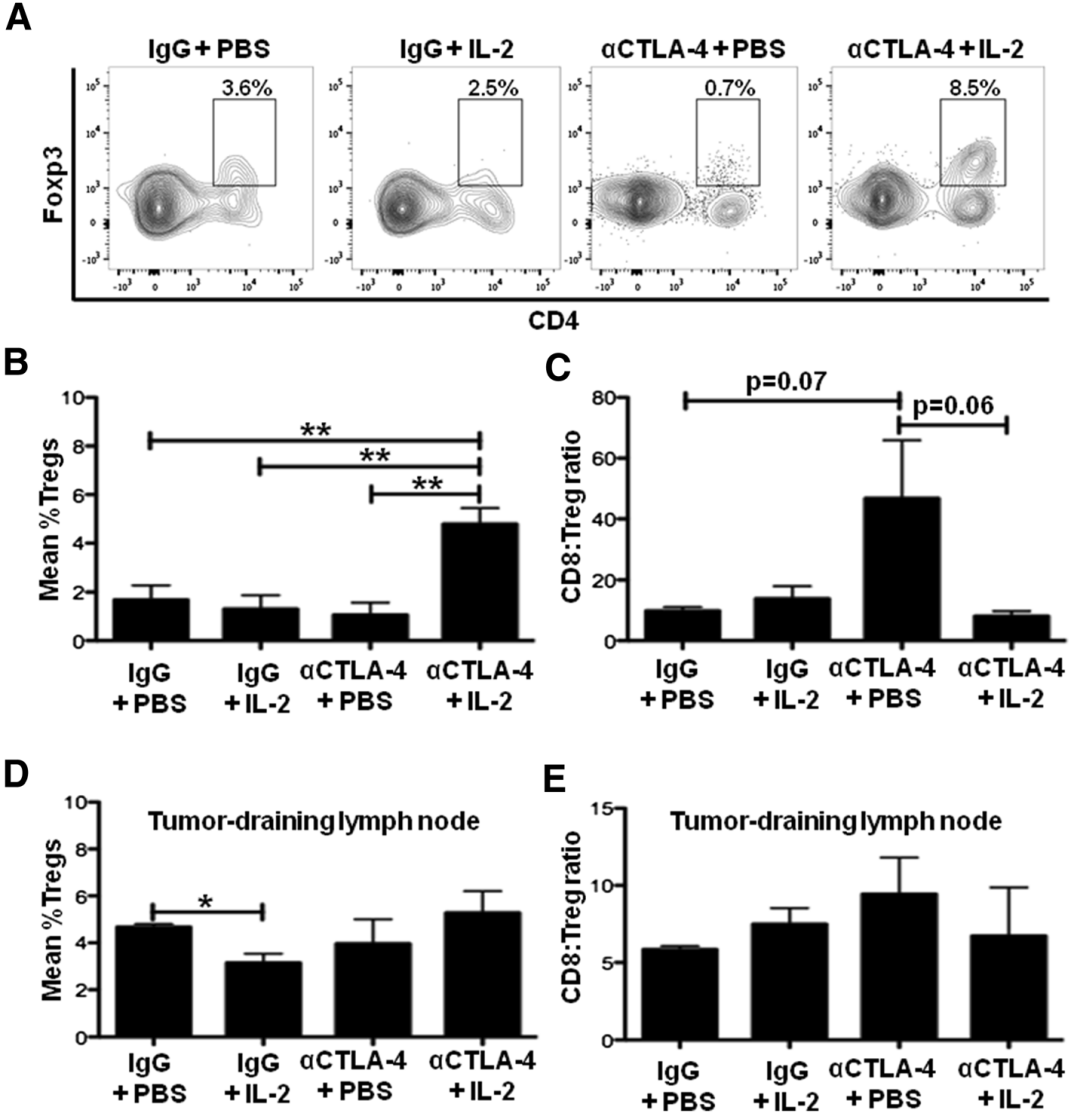
Combination IL-2 and CTLA-4 blockade immunotherapy increases the proportion of tumor-infiltrating Tregs. **(A)** Flow cytometry plots of tumors dissected at day 14 and analyzed for regulatory CD4 + Foxp3+ T cells (Tregs; of CD45 + CD3+ cells within the tumor) by flow cytometry from the experiment described in Figure 1A. **(B)** Cumulative graph showing mean percent of Tregs from experiment in **(A)**. **(C)** Ratio of CD8+ T cells to Tregs in the tumor from experiment in **(A)**. **(D)** Cumulative graph showing mean percent of Tregs from the tumor-draining lymph nodes from experiment in **(A)**. **(E)** Ratio of CD8+ T cells to Tregs in the tumor-draining lymph node from experiment in **(A)**. Cumulative figures are from at least three independent experiments (with 3–5 mice per group in each experiment). *P < 0.05, **P < 0.01, ns = not significant.

Regulatory T cells (Tregs) promote tumor growth through the suppression of anti-tumor immune responses. Tregs suppress CD8+ T cell and NK cell responses specifically through CTLA-4 [35] and by acting as IL-2 sinks through expression of high levels of the high affinity IL-2 receptor, CD25 [36]. Thus, we determined whether combination IL-2 and CTLA-4 blockade immunotherapy affects Tregs in the tumor. While CTLA-4 blockade alone and IL-2 alone did not increase the proportion of Tregs (among CD45 + CD3+ cells) within the tumor compared to the control treatment (1% and 2% versus 2%, p > 0.05 for both, respectively), the combination of these immunotherapies significantly increased Tregs among the immune infiltrate compared to the control treatment (5% versus 2%, respectively, p < 0.01) (Figure 4A, B). These findings demonstrate that the improved responses observed with combination IL-2 and CTLA-4 blockade immunotherapy compared to either monotherapy are associated, unexpectedly, with an increase in the proportion of Tregs in the tumor.

The efficacy of immunotherapies has been previously attributed to their ability to increase the proportion of CD8+ T cells to Tregs [31]; therefore, we determined this ratio within the tumor. We observed a trend towards an increased ratio of CD8+ T cells (of CD45+ cells) to Tregs (of CD45+ cells) in the CTLA-4 blockade monotherapy group compared to the combination IL-2 and CTLA-4 blockade immunotherapy group (47 versus 8, respectively, p = 0.06) and compared to the control group (47 versus 10, respectively, p = 0.07) (Figure 4C). However, we found no statistically significant differences in the ratio of CD8+ T cells to Tregs when comparing the combination IL-2 and CTLA-4 blockade immunotherapy group to any other group (p > 0.05 for all comparisons) (Figure 4C and data not shown). These data suggest that the CD8+ T cell to Treg ratio does not correlate with the improved anti-tumor responses observed with combination IL-2 and CTLA-4 blockade immunotherapy compared to either monotherapy in this model.

To determine whether the observed differences and trends in the proportion of Tregs and the ratio of CD8+ T cells to Tregs was confined to the tumor microenvironment, we determined these measures within the tumor-draining lymph nodes and spleen. Here, we observed no differences or trends in either the proportion of Tregs or the ratio of CD8+ T cells to Tregs with the monotherapies or the combination IL-2 and CTLA-4 blockade in the tumor-draining lymph nodes (in all groups Tregs constituted 3 to 5% of CD45+ cells, p > 0.05 for all comparisons; and in all groups CD8:Treg ratios were 6 to 9, p > 0.05 for all comparisons) (Figure 4D, E) or in the spleen (data not shown). These data suggest that the observed differences and trends in the proportion of Tregs and the ratio of CD8+ T cells to Tregs in the tumor-draining lymph nodes and spleen do not parallel those found in the tumor microenvironment.

### CTLA-4 blockade increases NK cells within the tumor infiltrate and IL-2 modulates NK cell differentiation status

**Figure 5:**
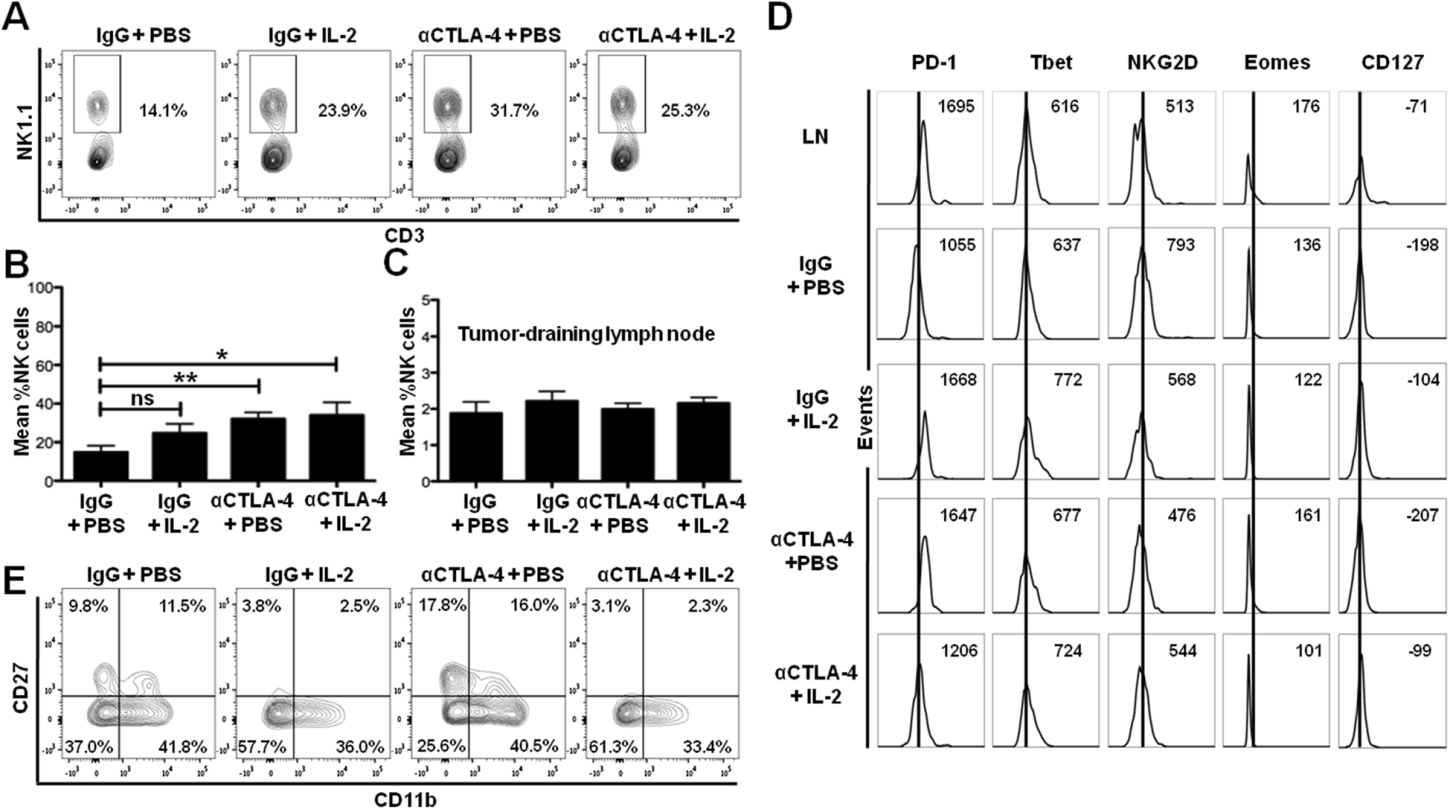
Combination IL-2 and CTLA-4 blockade immunotherapy increases the proportion and changes the differentiation of tumor-infiltrating NK cells. **(A)** Flow cytometry plots of tumors dissected at day 14 and analyzed for the proportion of NK cells (of CD45 + CD3- cells within the tumor) by flow cytometry from the experiment described in Figure 1A. **(B)** Cumulative graph showing mean percent of NK cells from experiment in **(A)**. **(C)** Cumulative graph showing mean percent of NK cells from the tumor-draining lymph nodes from experiment in **(A)**. **(D)** Representative histograms showing expression of cell markers by NK cells in the tumor and from a representative tumor-draining lymph node (LN). Numbers represent mean fluorescence intensity (MFI). **(E)** Flow cytometry plots showing expression of CD27 and CD11b on tumor-infiltrating NK cells from experiment in **(A)**. Cumulative figures are from at least three independent experiments (with 3–5 mice per group in each experiment). *P < 0.05, **P < 0.01, ns = not significant.

Since the therapeutic activity of the combination treatment did not depend on the CD8+ T cell:Treg ratio, we sought to determine if NK cells were involved in the antitumor activity. CTLA-4 blockade alone and in combination with IL-2 resulted in an increase in the proportion of NK cells among the non-T cell (CD45 + CD3-) tumor immune infiltrate compared to the control (32% and 34% versus 15%, p < 0.01 and p < 0.05, respectively) (Figure 5A,B). There were no differences in NK cell infiltration between the CTLA-4 blockade only and the combination IL-2 and CTLA-4 blockade treatment groups (32% versus 34%, respectively, p > 0.05) (Figure 5A,B). As with our observation for CD45+ cells, CD8+ T cells, and Tregs, the increase in the proportion of NK cells was not observed in the tumor-draining lymph nodes (in all groups NK cells constituted approximately 2% of CD45+ cells, p > 0.5 for all comparisons) (Figure 5C) or spleen (data not shown).

To determine whether the activation status of NK cells correlates with the anti-tumor immune responses we observed (Figure 1B,C), we determined the expression of well-described NK cell markers. There were no significant differences in tumor-infiltrating NK cell activation markers PD-1, Tbet, NKG2D, Eomes, and CD127 in the IL-2 or CTLA-4 blockade monotherapy or combination immunotherapy treatment groups versus the control group (Figure 5D). Because IL-2 has been reported to increase the proportion of immature NK cells [37] and regulate NK cell maturation, as determined by expression of CD27 and CD11b [38], we determined the effects of combination IL-2 and CTLA-4 blockade immunotherapy on the maturation status of NK cells within the tumor. Treatment with IL-2 alone and in combination with CTLA-4 blockade resulted in reduced expression of CD27 and CD11b compared to the control (3% and 2% versus 12%, p < 0.05 for both, respectively) on NK cells suggesting a less differentiated phenotype (Figure 5E). CTLA-4 blockade had no effect on NK cell maturation compared to the control group (16% versus 12%, respectively, p > 0.05) (Figure 5E). These results demonstrate that CTLA-4 blockade and IL-2 work in combination to increase the proportion of NK cells in the tumor infiltrate (via the effects of CTLA-4 blockade) and to promote a less differentiated NK cell population (via the effects of IL-2) within the tumor microenvironment.

Since one possible mechanism through which NK cells might mediate anti-tumor activity is to delete MHC class I negative tumor cells, and because melanoma has been reported to evade immune detection through loss of MHC class I expression, we evaluated the level of MHC class I on the B16 tumor cells after *in vivo* challenge. We observed that over 92% Melan-A+ cells in the tumor demonstrated surface expression of MHC class I molecule H-2K^b^ (Additional file 1: Figure S1). This finding suggests that the importance on NK cells in the context of combination IL-2 and CTLA-4 blockade immunotherapy is not based on loss of MHC class I expression on tumor cells.

### CD8+ T cells and NK cells are necessary for the efficacy of combination IL-2 and CTLA-4 blockade immunotherapy

**Figure 6:**
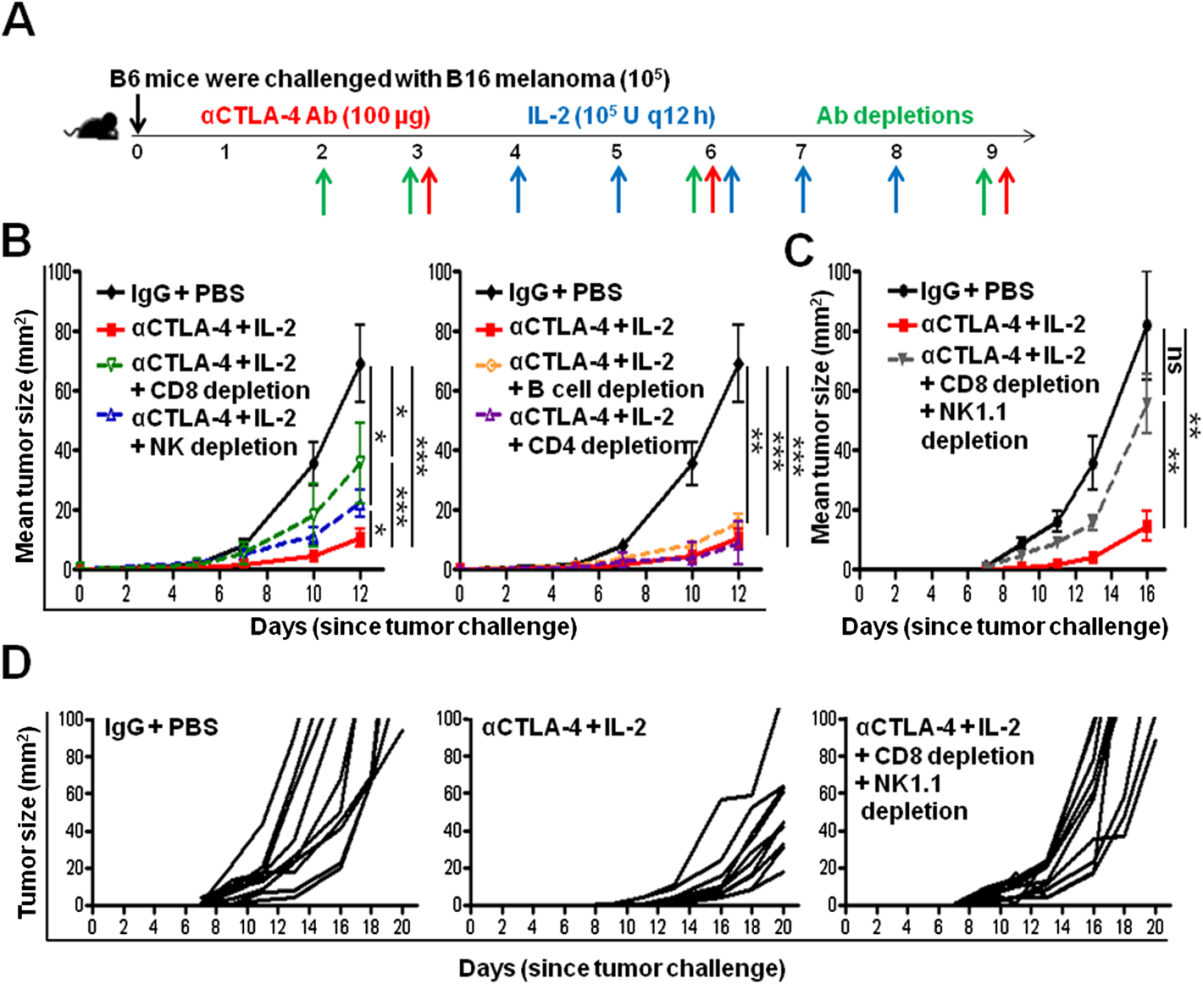
NK and CD8+ T cells are necessary for combination IL-2 and CTLA-4 blockade immunotherapy-mediated effects. **(A)** Schematic of the experimental design. **(B)** Cumulative graph of the mean tumor size (mm^2^) per group after CD8 or NK cell depletion (left panel) and B cell or CD4+ cell depletion (right panel) from the experiment described in **(A)**. **(C)** Cumulative graph of the mean tumor size (mm^2^) per group after combination CD8 and NK cell depletion from the experiment described in **(A)**. **(D)** Tumor size (mm^2^) of individual mice in each group from experiment described in **(C)**. Four to ten mice were included in each group. Graphs represent one experiment of three conducted with similar results. *P < 0.05, **P < 0.001, ***P < 0.001, ns = not significant.

NK cells can contribute to anti-tumor immune responses against B16 melanoma, as reported for various other types of combination immunotherapy approaches [39-41]. Thus, to determine if NK cells were involved in the anti-tumor activity of IL-2 and CTLA-4 blockade treatment, we repeated the tumor studies in Figure 1 in the absence of NK cell and/or or CD8+ T cells (Figure 6A). Individual depletion of CD8+ T cells or NK cells partially reduced the overall efficacy of the combination immunotherapy compared to treatment in immune competent mice (36 mm^2^ and 22 mm^2^ versus 11 mm^2^, p < 0.05 and p < 0.001, respectively, at day 12) (Figure 6B, left panel). However, depletion of either subset individually was not sufficient to completely ablate therapeutic efficacy compared to the control (36 mm^2^ and 22 mm^2^ versus 69 mm^2^, p < 0.05 for both, respectively, at day 12) (Figure 6B, left panel). Depletion of neither CD4 nor B cells had any effect on the efficacy of the combination IL-2 and CTLA-4 blockade immunotherapy compared to no depletion (9 mm^2^ and 16 mm^2^ versus 11 mm^2^, p > 0.05 for both, respectively, at day 12) (Figure 6B, right panel). When both CD8+ T cells and NK cells were depleted concurrently during the course of combination IL-2 and CTLA-4 blockade immunotherapy, therapeutic responses were abrogated compared to no depletion (82 mm^2^ versus 14 mm^2^, respectively, p < 0.05, at day 16) and tumor growth resembled that of the control group when both CD8+ T cells and NK cells were depleted concurrently (82 mm^2^ versus 55 mm^2^, respectively, p > 0.05, at day 16) (Figure 6C,D). Further depletion of both CD8 T cells and NK cells concurrently reduced the therapeutic response significantly compared to either CD8 or NK cell depletion alone (p < 0.01 for both comparisons). This demonstrates that both CD8+ T cells and NK cells are necessary for the therapeutic effectiveness of combination IL-2 and CTLA-4 blockade immunotherapy.

## Conclusions

High dose IL-2 and CTLA-4 blockade have demonstrated clinical success in the treatment of advanced melanoma although the exact mechanism of their anti-tumor activity is not completely defined [14,12,25]. CTLA-4 suppresses immune responses by limiting co-stimulation of T cells through binding of CD80 and CD86 [42] and reducing T cell receptor signaling [42]. In contrast, IL-2 promotes effector responses, regulates differentiation, and mitigates exhaustion of both CD8+ T cells and NK cells. Previous studies have shown that CTLA-4 blockade can mediate therapeutic activity by enhancing T cell responses as a result of increased endogenous expression of local IL-2 [29,43]. Therefore, we sought to evaluate the combination of CTLA-4 blockade and high-dose exogenous, recombinant IL-2 with the hypothesis that CTLA-4 blockade would prevent immune suppression while administration of IL-2 would synergistically enhance CD8+ T cell and NK cell responses. This combination immunotherapy did improve therapeutic responses over either CTLA-4 blockade or IL-2 monotherapy (Figure 1) against the poorly immunogenic B16 melanoma. CD8+ T cells and NK cells were both necessary for the therapeutic effect of the combination immunotherapy as witnessed by partial reductions in efficacy when either CD8+ T cells or NK cells were depleted individually (Figure 6). The potential for improved clinical outcomes has also been suggested by a small dose escalation clinical trial that tested the combination of high-dose IL-2 (720,000 IU/kg) with increasing doses of ipilimumab, a humanized monoclonal antibody that blocks CTLA-4 (range 0.1 - 3.0 mg/kg) in patients with advanced melanoma [44]. In this trial an initial objective response rate of 22% was reported. In further follow-up, however, the overall response rate improved to 28% with a remarkable 17% of patients achieving a complete response [45]. This is impressive considering the delayed responses often observed with ipilimumab and the fact that some patients received very low doses of ipilimumab. Our data reported here adds additional support to further clinical studies of combination IL-2 and ipilimumab in patients with metastatic melanoma.

In this study, we found that CTLA-4 blockade promotes immune infiltration into the tumor microenvironment. In particular, CTLA-4 blockade increased the relative proportion of CD8+ T cells and NK cells among tumor-infiltrating immune cells. In line with previous publications that have used CTLA-4 blockade as a monotherapy [31], we observed that this preferential infiltration of CD8+ T cells and NK cells resulted in a trend towards an increased ratio of CD8+ T cells to Tregs (Figure 4C). This ratio has been suggested as an indicator of therapeutic response given the anti-tumor cytotoxicity function of CD8+ T cells as opposed to the suppressive actions of Tregs [31]. In our model this ratio was not highly significant and was not increased when CTLA-4 blockade was combined with IL-2. This may be related to how the ratio was calculated as we standardized the number of T cells to the CD45+ cell population, whereas others have standardized to all live cells. Further, there is evidence that some Foxp3+ CD4+ T cells (particularly those that are FoxP3^lo^) may not be suppressive; therefore, the differences in Foxp3+ CD4+ T cells may not indicate an increase in suppressive regulatory CD4+ T cells [46]. Future studies will explore the suppressive ability of these CD4+ Foxp3+ T cells in the context of the combination immunotherapy. Another explanation may be that other mechanisms may mediate tumor rejection in our model, including the accumulation of less differentiated/exhausted NK cells, which were increased in the tumor microenvironment of mice treated with the combination immunotherapy. Since IL-2 is known to expand Tregs it may not be surprising that therapeutic effects were not associated with a shift in the CD8:Treg ratio with this therapeutic combination. Studies of immunosurveillance in mice have suggested that complete tumor elimination is dependent on induction of both innate and adaptive immune responses. Thus, IL-2-mediated expansion of NK cells, which was necessary for the therapeutic activity observed with combination treatment, may override or supplement a decrease in Tregs. The necessity of NK cells for therapeutic activity is in line with other studies highlighting a contributing role of NK cells in immunotherapy, potentially through direct cytoxicity or IFN-γ production [41,40,39]. Our results are in agreement with these studies and supports the potential role of NK cells in immunotherapy especially in weakly immunogenic tumors.

Although other mechanisms may contribute to the therapeutic responses, our data suggests that CTLA-4 blockade may help recruit NK cells to the tumor microenvironment while IL-2 (alone and in combination with CTLA-4 blockade) altered the maturation of tumor-infiltrating NK cells. NK cells are thought to progress through maturation marked by expression of CD27 and CD11b. NK cells mature first by upregulating CD27 then CD11b [38]. IL-2 either as a monotherapy or in combination with CTLA-4 blockade increased the proportion of less fully differentiated (CD27-CD11b-) NK cells as compared with the exhausted (CD11b+) NK cells within the tumor observed with CTLA-4 blockade alone. These results, when considered with the data demonstrating that NK cells participate in mediating the therapeutic efficacy of the combination immunotherapy, suggest that IL-2 has a role in the NK cell portion of this response, potentially through reducing NK cell exhaustion or expanding non-exhausted NK cells. NK cells may also cooperate with CD8+ T cells by targeting MHC class I-negative and -positive tumor cells, respectively. This is unlikely the case in this model as MHC class I expression was quite robust following in vivo tumor transplantation. Since NKG2D has been shown to help mediate tumor elimination during immunosurveillance of spontaneous tumors, it is also possible that less differentiated NK cells recruited to the microenvironment may utilize NKG2D to help mediate tumor immunotherapy. Since NK cell recruitment may also result in release of local interferon-gamma, this might also help improve CD8+ T cell function by up-regulating tumor antigens, MHC Class I and enhanced cytotoxic activity.

In summary, combination immunotherapy with high-dose IL-2 and CTLA-4 blockade, compared to either monotherapy alone, improves therapeutic responses in the poorly immunogenic B16 murine melanoma model. This response is dependent on recruitment of both effector CD8+ T cells and early activated NK cells to the tumor microenvironment. Future studies will focus on the antigen-specificity of the CD8+ T cell responses and attempts to better understand how NK cells are contributing to the anti-tumor activity. In the interim, the data support clinical development of IL-2 and CTLA-4 blockade as a rational combination immunotherapy for patients with melanoma. A clinical trial testing the combination of high-dose IL-2 and ipilimumab is planned, including an assessment of CD8+ T and NK cell responses, and could represent a new treatment strategy for patients with melanoma.

## Methods

### Mice and cells

C57BL/6 (B6) age 6–8 weeks were purchased from The Jackson Laboratory (Bar Harbor, ME) and housed in a specific pathogen-free facility at Rush University Medical Center. All melanoma cells were cultured in RPMI supplemented with 10% heat-inactivated fetal bovine serum (Atlanta Biologicals, Flowery Branch, GA), 2 mM L-glutamine (Mediatech, Manassas, VA), and 1% penicillin/streptomycin (Mediatech). Animal procedures and protocols were performed in accordance with Rush University Medical Center Institutional Animal Care and Use Committee guidelines.

### Tumor challenge

Mice were anesthetized with isoflurane and challenged with B16-F10 melanoma via intradermal injection with 100,000-120,000 cells in the right shaved flank (5–10 mice per group, with each experiment repeated multiple times with similar results), as previously described [47]. Tumor area (length x width) was measured every two to three days until death of the animal or until tumors reached 100 mm^2^, when animals were sacrificed as per institutional protocols. Primary outcomes included tumor size and overall survival. In some experiments, tumors, spleens, and tumor-draining inguinal lymph nodes were obtained.

### Flow cytometry and staining

Cell staining data were collected with the Canto II flow cytometer (BD, Franklin Lakes, NJ) and analyzed with FlowJo software (Tree Star, Ashland, OR). Gating on live, singlet, non-debris lymphocytes was performed using LIVE/DEAD staining, forward scatter area (FSC-A) versus side scatter area (SSC-A), forward scatter width (FSC-W) versus side scatter width (SSC-W), FSC-A versus forward scatter height (FSC-H), and SSC-A versus side scatter height (SSC-H) plots, as previously described [48]. All non-melanoma antibodies were purchased from eBiosciences (San Diego, CA). Intratumoral lymphocytes were delineated through *in vivo* retroorbital injection of anti-CD45 (FITC) three minutes prior to tumor resection to label leukocytes restricted to the vasculature, as previously described [49]. MHC Class I staining was performed by gating on Melan-A-positive cells (antibody purchased from Santa Cruz [Dallas, TX] followed by staining for surface expression of H-2K^b^. For immunofluorescence microscopy staining tissues were treated and images were acquired with an automated Leica DM5500B microscope, as described previously [50].

### Treatments and antibody depletions

Mice were treated with 100 μg CTLA-4 antibody blockade (9H10) from BioXcell (West Lebanon, NH) administered by intraperitoneal (i.p.) injection on days 3, 6, and 9 post tumor challenge or with 100 μg of the appropriate IgG clones (BioXcell). Recombinant human IL-2 (100,000 units resuspended in PBS delivered by i.p. injection; Prometheus Laboratories, Inc., San Diego, CA) or PBS control was administered every 12 hours on days 4–8. CD8+ T cells, NK cells, CD4+ T cells, and B cells were depleted with 250 μg of anti-CD8 (53–6.72), anti-NK1.1(PK136), anti-CD4 (GK1.5), and anti-CD19 (1D3) purchased from BioXcell, respectively, on days 2, 3, 6, and 9. Similarly, 250 μg of IgG (appropriate clones; BioXcell) were injected as a control.

### Statistical analysis

Student’s t test (two-tailed) and logrank test were used for comparisons of data and survival curves, respectively, using GraphPad Prism software (v4.0, GraphPad Software, Inc., La Jolla, CA). A p value of less than 0.05 was considered to denote statistically significant comparisons.

## Additional file

Additional file 1: Figure S1. B16 melanoma cells express high level of MHC class I. Flow cytometric histogram of H-2k^b^ expression on Melan-A+ cells from a tumor dissected on day 14 from a B6 mouse. Blue = isotype staining, Red = H-2k^b^ staining. Numbers listed correspond to MFI (mean flourescence intensity).
