# Supplementary figures and images for: NK cells and CD8+ T cells cooperate to improve therapeutic responses in melanoma treated with interleukin-2 (IL-2) and CTLA-4 blockade
Source: J Immunother Cancer. 2015 May 19;3:18. doi: 10.1186/s40425-015-0063-3 (PMC4437746; doi:10.1186/s40425-015-0063-3)

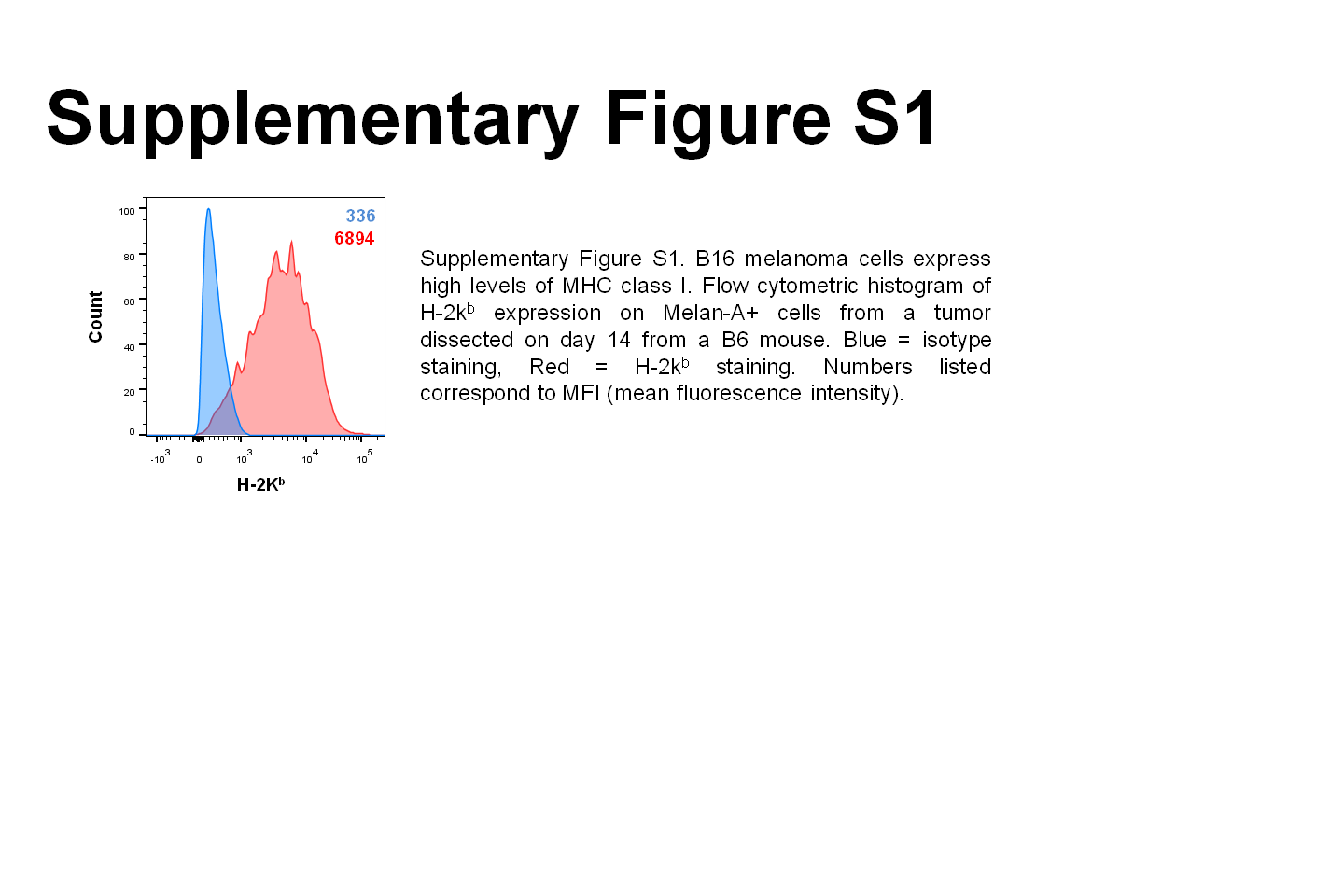

Supplement: Additional file 1: Figure S1. — B16 melanoma cells express high level of MHC class I. Flow cytometric histogram of H-2kb expression on Melan-A+ cells from a tumor dissected on day 14 from a B6 mouse. Blue = isotype staining, Red = H-2kb staining. Numbers listed correspond to MFI (mean flourescence intensity). [file 40425_2015_63_MOESM1_ESM.tif]
